# The Magnetic Urtext: Restoration as Music Interpretation

**DOI:** 10.3389/fpsyg.2022.844009

**Published:** 2022-04-20

**Authors:** Sergio Canazza, Emery Schubert, Anthony Chmiel, Niccolò Pretto, Antonio Rodà

**Affiliations:** ^1^Centro di Sonologia Computazionale, Department of Information Engineering, University of Padova, Padua, Italy; ^2^Empirical Musicology Laboratory, School of the Arts and Media, University of New South Wales, Sydney, NSW, Australia; ^3^The MARCS Institute for Brain, Behavior and Development, Western Sydney University, Sydney, NSW, Australia

**Keywords:** audio document preservation, re-interpretation, electronic tape music, archiving, reel to reel recording, restoration, urtext, heritage

## Abstract

This paper discusses *historical-critical thought* to address the problems of restoration and preservation of tape music, proposing viable solutions to the matter of digitizing the historically valuable data that exists on and is represented by magnetic tapes. A detailed program of research and restoration and some software for helping in creation of critical editions of the musical works are proposed. We also present some of the issues and controversies that must be considered and approaches we have applied in the preservation of tape music, highlighting how these *interpretations* can impact later performances (playback) of these tape documents. Fundamentally, we argue that the act of tape music restoration has a parallel with the interpretation of the “Urtext score” in the performance of music from the Common Practice Era.

## Introduction

The enormous industrial scale production of sound recordings (e.g., wax cylinders, phonographic discs, audio cassettes), in addition to an incalculable quantity of audio documents produced outside the industrial process of recording, has progressively become an integral part of the documentary heritage of the twentieth century (Bressan and Canazza, 2013). Frozen in a mass of recordings made in the last century by analog devices, these events, performances, electronic tape music compositions (hence “tape music”), and soundtracks can now survive the degradation of their carriers (the material on which the audio coded documents are stored, see Bressan et al., 2016) and can be duplicated by digitizing the audio signals, which gives them a representation that can be losslessly conveyed in the new media environment (Canazza et al., 2015).

We introduce a *historical-critical* approach to address the problems of preservation, edition, and access of documents according to a plurality of *interpretative* choices. This is guided by the knowledge of the internal history of the document and the study of both the material and the technological conditions that produced it and the contexts in which the document was produced. Such considerations must be carried out on the basis of a genuinely interdisciplinary approach, considering the restoration as a philological operation of *constitutio textus* (i.e., in the textual criticism: to produce a text as close as possible to the original; Maas, 1958). This paper will examine: (1) Tape Music (section “Tape Music”)—setting up the significance of restoring audio reel to reel compositions from a historical perspective, to demonstrate that there exists precious source material that is at risk of being a lost heritage; (2) Tape Music preservation (section “Tape Music Preservation”)—detailing the technical problem of deterioration of analog magnetic tape; (3) Restoration as reinterpretation (section “Restoration as *Reinterpretation”*)—(a) introducing the (somewhat counterintuitive) need to alter the source signal to allow faithful reproduction; and (b) discussing their implications for the digitization processes, to which we then turn our attention, and (4) (section “Conclusion”) proposing viable solutions to the matter of digitizing the historically valuable data that exist on and are represented by these magnetic tapes.

## Tape Music

The quest for authentic reproduction of the *great compositions* (Taruskin, 2010) ushered in the concept of the urtext (*original* and coming to mean *originally intended*) score. Despite the limitations of the urtext idea in printed scores (Feder, 2011) analogy with the current question is striking. By considering additional technical and sociological factors, the historically significant audio tape is importantly different to the music score in that the music documentation *is* the sound world source that the creator inhabited. If enough is known about the reproduction of the source (e.g., the reproductive equipment, the ambience of the typical playback venue) then the goal of obtaining access to the *authenticity* of the creative act and output, directly from the document, becomes feasible, in contrast to music notation of the past. From this point of view the magnetic tape is undoubtedly an urtext (see, Somfai, 1981), and realizing this, the urtext concept is practicable and, we shall argue, feasible.

Our project is concerned with pieces of music that come into existence as a result of placement of audio on the sound recording medium. Unlike recordings of live instrumental musicians (which have their own set of problems, e.g., Fabian, 2003), the music that concerns us here is not recorded on a podium to be later stored and reproduced, but it is born with the help of electronic valves, transistors, and the like; it “exists” only on magnetic tapes (in the case we are considering) and can be listened to with loudspeakers. Such techniques of composition became viable with the invention of magnetic tape sound recording technologies where direct human manipulation and (acoustic-)electromagnetic treatment of the recording medium was possible. And so, it attracted the interest of the most important experimental and avant-garde creative minds of the mid-twentieth century in Europe, including Edgard Varése (1883–1965), Olivier Messiaen (1908–1992), Iannis Xenakis (1922–2001), Luigi Nono (1924–1990), Luciano Berio (1925–2003), Pierre Boulez (1925–2016), and Karlheinz Stockhausen (1928–2007).

In the United States we can see an extreme example of how the physical interaction with the recording medium was also part of the artistic process, in John Cage’s (1912–1992) four and a half minute (at 15 ips tape playback speed) work *Williams Mix* (1952). The creative process involved preparing “minutely and obliquely cut pieces of magnetic audiotape, chosen and spliced together through chance operations from a stack of 500 to 600 recorded sounds in six categories—city sounds, country sounds, electronic sounds, manually produced sounds (including the literature of music) and wind produced sounds (including songs) and small sounds requiring application to be heard with others” (Kahn, 1999, p. 112). From a philological perspective this process, and the state of the magnetic tape after the process, must also be considered part of the artwork, making it important to consider the visuo-spatial-tactile-olfactory world in restoration (re-interpretation), not just the sound world encoded on the tape (Benjamin, 1955).

## Tape Music Preservation

The preservation and restoration of historically significant tape music recordings is an urgent aspect of musicology research. Moreover, as this issue is linked to philological, musicological, and information engineering areas, it is a central concern for performance science, yet is often overlooked.

**FIGURE 1:**
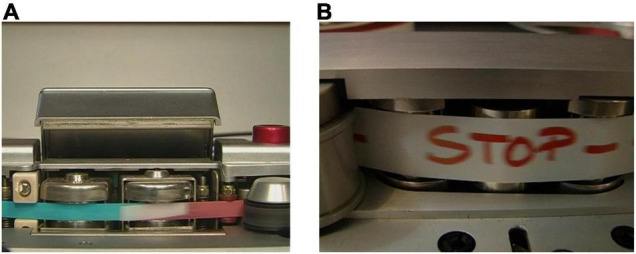
**(A)** Spliced tape; **(B)** signs by the composer on the tape.

As composers manipulate tapes with cutting and splicing techniques (see Figure 1A) as well as marks on the tape (Figure 1B), for which the analog magnetic tapes are the only available documentation, we propose that the tape itself is an artwork. Analog tapes deteriorate under normal storage conditions, and playback of the tape further hastens deterioration. Conversely, indefinite storage in hermetically sealed, temperature-controlled environments renders the material inaccessible.

Analog-to-digital conversion is an indispensable mode of preserving audio documents, as archived tape music recordings are often the only reference material, and without careful preservation and restorative intervention these musical works would be permanently lost. The use of specific playback technology is further tied to the compositional process, as reproduction on modern equipment may diverge considerably from the sound world intended by the composer. Thus, the tape is an integral part of the music performance that provides artistic value as well as significance as a historical document. To save valuable historical recordings, the audio signal and other relevant information (such as metadata and contextual information) can be extracted from the source audio document and transferred onto another medium such as a digital storage system (a remediation process).

An example of the complexity of restorative choices in the field of electronic music on tape is given by the work *Y entonces comprendió* (1970) by Luigi Nono. In this work the composer re-records an existing tape source (containing Fidel Castro’s voice, reciting the words of Che Guevara) onto disc; this leads to a noticeably different timbre between the sources. In these hybrid works the relationship between tape and live interpretation favors the permanence of spontaneity, which increases the weight of the source material, full of unpredictability and originality, with respect to the preserved product. Here, therefore, there is the choice of whether or not the restoration should leave a trace of the source: voice and sounds recorded on a disc are linked to the noise of the disc, which is different from that of the tape. Different noises produced by a continuous transfer on different media leave a trace of the system that produced the document.

## Restoration as *Reinterpretation*

A tape music document is related to the playback system and therefore a musicologist (and a live electronics performer) must use technical (analog and then digital) devices for both music playback and sound texture analysis. Two factors are combined: human attention (that can introduce errors, i.e., *noise*) and the imperfection of the machine (that introduces noise, i.e., *errors*). The interpretation of the tape requires systemic reconstruction in the world of experimental electronic music praxis (e.g., of the interactions between the composer, sound engineer, and audio technology used). Several factors impact the reconstruction, showing parallels with traditional music scores, including: (1) knowledge of the recording system, (2) exact identification of the recording format, (3) metadata transmitted by the whole documentary unit, (4) track labels and numbers, (5) editing operations by the technician and the composer, (6) writings on the tape (e.g., markings by the composer for synchronization of the performance, as in Figure 1B), and (7) the composer’s performance prescriptions. The remediation process is not neutral and opens the philological issue of document authenticity. Removal, enhancement, or compensation actions can lead to different interpretations.

Several *interpretive* choices need to be made (Brandi, 1977), which can be particularly complex in the field of tape music. A philology of sound documents is urgently needed because of the apparent simplicity with which the transfer from the analog to the digital domain can be carried out, coupled with the potential for inaccuracy here. The audio document includes information related to its realization process, which is significant for the audio content transferring (Bressan and Canazza, 2013). Specifically, (a) the physical-chemical structure of the carrier; the technology, the production system (acoustic, electroacoustic, magnetic), the recording format (total number of tracks, playback speed, etc.); (b) the primary (useful) information relating to the message contained in the recording; (c) the secondary (ancillary) information: signals characterizing the recording system; (d) the playback and listening system; (e) metadata (labels, notes on the case, etc.); (f) the history of its transmission (types of archiving, duplications, digitization, playback dates/locations, etc.).

The Centro di Sonologia Computazionale (CSC) of University of Padova has defined a rigorous scientific protocol for different interpretative approaches, during several restoration projects involving the most important European music archives (Canazza and De Poli, 2020): in the following we describe two of them, used in restoration projects related to tape music.

(1) The ***archivist’s*** interpretative choice needs to consider the information stored in the audio document as an artifact. It focuses on the document physiognomy and aims to preserve the documentary unit. Its bibliographic equivalent is the diplomatic copy or *facsimile*. The format detection and the choice of the playback system are essential. Restoration processing is considerably aided by good documentation, and amenable to restoring the functionality of the carrier (splicing at the breaking points, hydration of the substrate to counteract hydrolysis, etc.; Bressan et al., 2016). Intentional endogenous alterations of the signal (equalization, noise reduction systems, and *compander*, e.g., DBX Type I) made in the recording process are compensated. This interpretative choice aims to preserve material characteristics of the document, and optimize similarity of the new digital medium with the original document.

The output of this interpretative choice is the *preservation master*, that includes high resolution uncompressed audio (96 kHz/24 bit) and contextual information, stored using a combination of textual, photographic and video documentation (Orio et al., 2009; Fantozzi et al., 2017; Pretto et al., 2019). Video of the tape flows along the head of the tape player during digitization. In addition to the contextual information of tape discontinuities (e.g., Figure 1A) and facilitating one’s potential to discriminate intentional from unintentional alterations, the video recording also offers the possibility to verify other irregularities in the playback speed of the tape which cause changes in frequency (i.e., wow or flutter). Photos report accurate information about labels, edition boxes and other attachments, as well as clearly visible magnetic tape (or—also—phonographic disc) corruptions. Finally, information that cannot be directly represented in digital format has to be thoroughly documented in the descriptive sheet (e.g., smell of a magnetic tape, which can indicate the presence of syndromes and olfactory characteristics like mold, vinegar odor, etc.).

**FIGURE 2:**
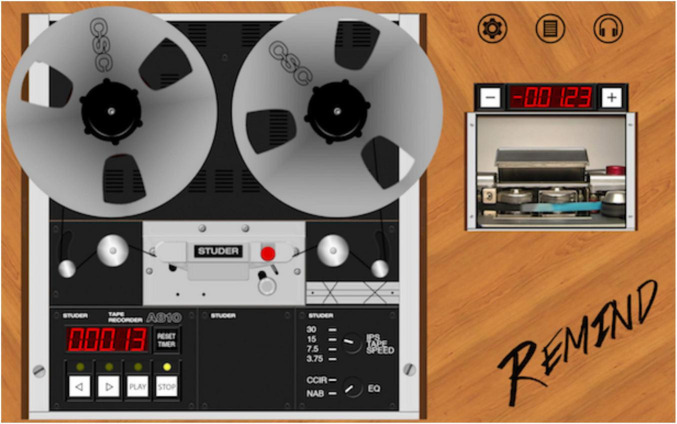
The main graphic user interface of the REMIND app. A video of the original tape is shown outside the body of the tape recorder (top right inset) to improve its readability.

The preservation master could be re-experienced by means of the software application REMIND (acronym for “Restoring the Experience: Mobile INterfaces for accessing Digitized recordings”) developed by a selection of the authors (Canazza et al., 2015), aimed at re-creating the experience of a phonograph or a reel-to-reel audio tape recorder (the focus of the present paper), including a complete set of metadata and contextual information (Figure 2 and Supplementary Figure 1 show the two graphic user interfaces of the app).

(2) The ***philological*** interpretative choice pays attention to (a) the fact that multiple versions of the same work can exist (referred to in philology as multiple *witnesses*), and the relationships between these versions (which can be created by collation of these witnesses *via* various means of audio digital process techniques; it is possible to examine these versions by comparison, or analysis by synthesis); (b) the audio equipment and techniques used in the recording phase; and (c) the compositional practice. The revived product is based on a single document, with a process that restores the audio to the technological state as close as possible to when it was first reproduced, along with the limitations of its contemporary technologies, rather than artificial enhancements or inauthentic degradation. If unintentional alterations exist (e.g., misalignment of the recording equipment), it is possible to subsequently compensate for the A/D conversion of the original signal. This interpretative choice aims to create historically informed *critical editions* including reports on *variant copies*, necessary for the reconstruction of the sound texture of each recording.

The 30-year experience that the authors have gained in the field of audio philology has resulted in the development of software CAP (Computer Aided audio Philology) that supports researchers in the processes of *recensio*, *collatio*, *eliminatio, codicum descriptorum*, and *stemma codicum* (West, 1973; Verde et al., 2018) (see main interface in Supplementary Figure 2). An operator inserts all the data, metadata, and contextual information in CAP, which creates the final critical edition of the musical work.

## Conclusion

Tape music from the 1940s to the 1980s is in urgent need of archiving because we are starting to lose the direct human, hardware and information links to those heritage sound worlds, which are critical for authentic and comprehensive archiving. Our philosophy of tape music archiving is that it is not solely a technical restorative act, but an artistically interpretive one, with some analogies to the historically informed performance of conventional music, where interpretation of the score is needed even if knowledge about performance practice is no longer fully available. In tape music restoration we do not have to retrace the *true* sound, but through an *interpretative* process of the materials we aim to safeguard the compositional and recording techniques of the time, such as in the two examples described in section “Restoration as *Reinterpretation*.”

The evolution from analog tape editing to computer programming poses a new and challenging task for the preservation of electronic music works, including the electronic music *born digital* production. Specifically, the rapid obsolescence of computer equipment and the loss of information on performance-compositional practice endanger the survival of this recent musical heritage. Therefore, progress in this area will need to focus on saving the original technological environment, developing a philology-informed hardware/software virtual emulator to replicate functions of obsolete audio technology, interpreting computer music composed using different programming languages, and systematically documenting primary witnesses (work versions) and composer experiences. Audio philology, specifically in the field of tape music, needs specially designed and developed software. Frameworks such as CAP, combined with algorithms capable of interpreting multichannel acoustic information (Salvati and Canazza, 2014) and phylogenetic techniques (Verde et al., 2018) will enable a process of preservation of the work and its reactivations, taking into account of the interpretative possibilities in its technological and physical-acoustic aspects.

## Data Availability Statement

The original contributions presented in the study are included in the article/Supplementary Material, further inquiries can be directed to the corresponding author/s.

## Author Contributions

SC, ES, and AR: conceptualization. SC and ES: writing—original draft preparation. AC and NP: writing—review and editing. NP: REMIND software. All authors have read and agreed to the published version of the manuscript.

## Conflict of Interest

The authors declare that the research was conducted in the absence of any commercial or financial relationships that could be construed as a potential conflict of interest.

## Publisher’s Note

All claims expressed in this article are solely those of the authors and do not necessarily represent those of their affiliated organizations, or those of the publisher, the editors and the reviewers. Any product that may be evaluated in this article, or claim that may be made by its manufacturer, is not guaranteed or endorsed by the publisher.
