# Supplementary material for: The Magnetic Urtext: Restoration as Music Interpretation
Source: Front Psychol. 2022 Apr 20;13:844009. doi: 10.3389/fpsyg.2022.844009 (PMC9067449; doi:10.3389/fpsyg.2022.844009)
Supplement: Supplementary file 1 [file Data_Sheet_1.pdf]

doi: 10.3389/fpsyg.2022.844009

Supplementary Material

The Magnetic Urtext: Restoration as Music Interpretation

1 Supplementary Figures

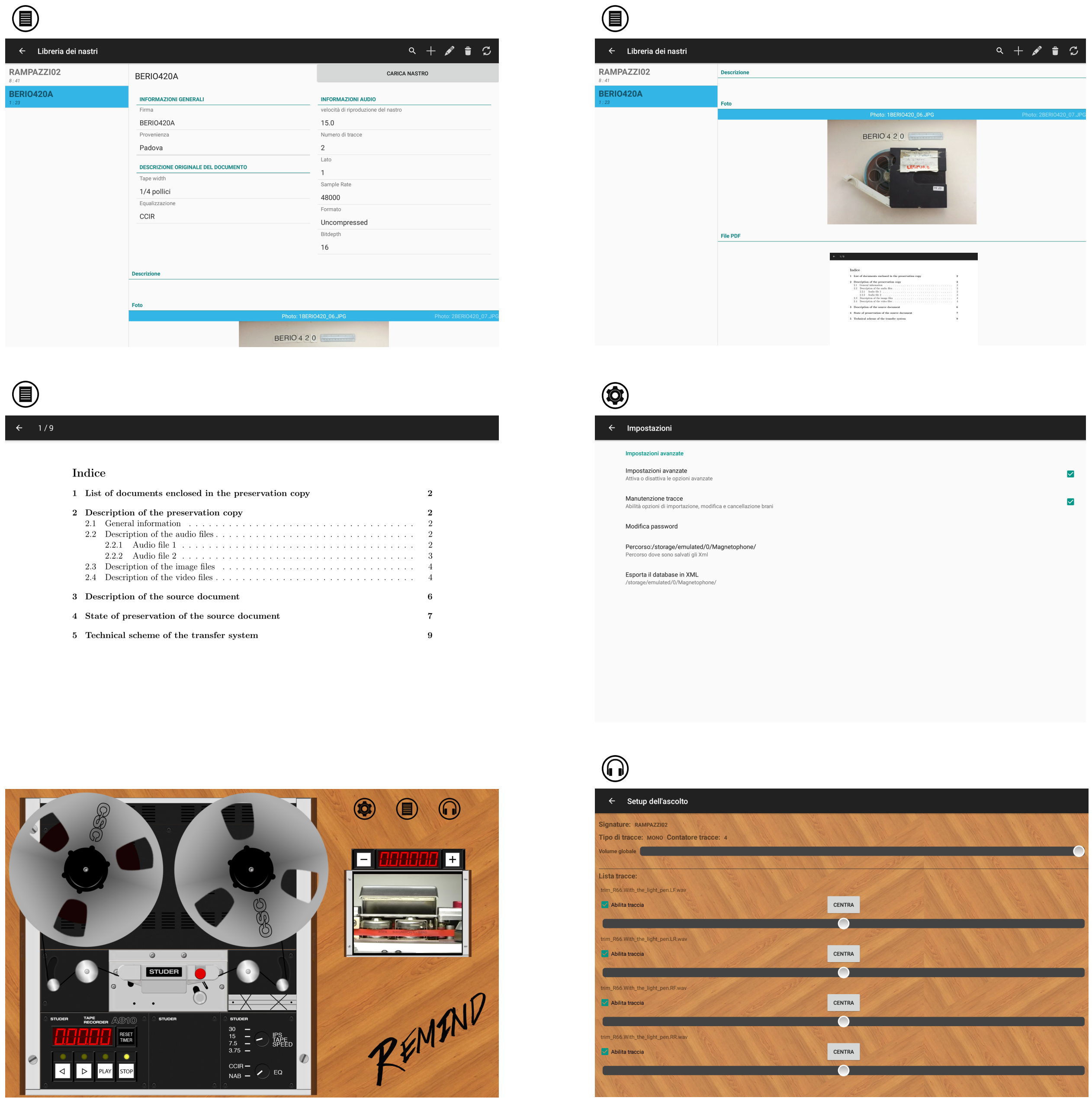

Supplementary Figure 1. The graphic user interface of the REMIND app related to the metadata of the preservation master. Text is set to Italian, for use by researchers at CSC, University of Padova.

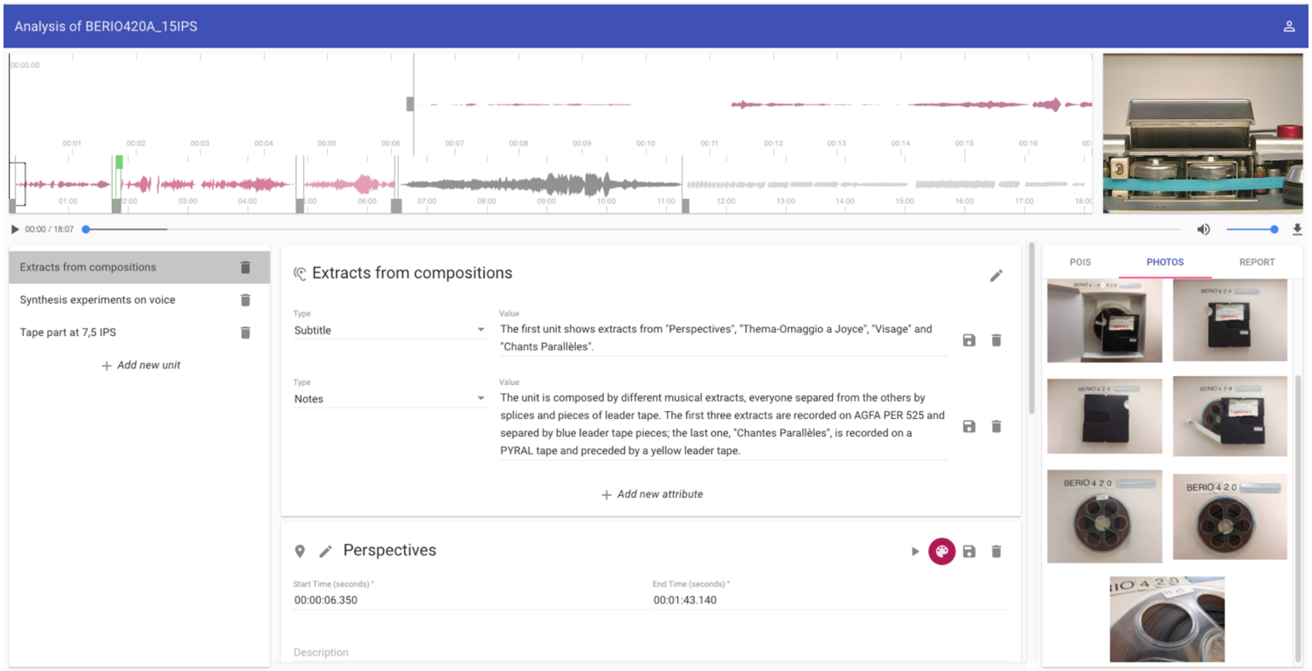

Supplementary Figure 2. The main interface of Computer Aided Audio Philology (CAP) software.
